# Challenges to the improvement of obstetric care in maternity hospitals of a large Brazilian city: an exploratory qualitative approach on contextual issues

**DOI:** 10.1186/s12884-018-2088-3

**Published:** 2018-11-26

**Authors:** Margareth Crisóstomo Portela, Sheyla Maria Lemos Lima, Lenice Gnocchi da Costa Reis, Mônica Martins, Emma-Louise Aveling

**Affiliations:** 1 Department of Health Administration and Planning, National School of Public Health, Oswaldo Cruz Foundation, Rio de Janeiro, RJ Brazil; 2 Department of Health Policy and Management, Harvard T.H. Chan School of Public Health, Boston, MA USA; 3 The Healthcare Improvement Studies Institute, University of Cambridge, Cambridge, UK

**Keywords:** Quality improvement, Obstetric care, Organizational context, Improvement science, Qualitative approach

## Abstract

**Background:**

Maternal morbidity and mortality are still serious public health concerns in Brazil, and access to quality obstetric care is one critical point of this problem. Despite efforts, obstetric care quality problems and sub-optimal/poor outcomes persist. The study aimed to identify contextual elements that would potentially affect the implementation of an obstetric care quality improvement intervention.

**Methods:**

A qualitative study was conducted in three public maternity hospitals of a large Brazilian city, with high annual volume of births and buy-in from high-level managers. Individual interviews with doctors and nurses were conducted from July to October 2015. Semi-structured interviews sought to explore teamwork, coordination and communication, and leadership, being open to capture other contextual elements that could emerge. Interviews were recorded and transcribed, and the categories of analysis were identified and updated based on the constant comparative method.

**Results:**

Twenty-seven interviews were carried out. Extra-organizational context concerning the dependence of the maternity hospitals on primary care units, responsible for antenatal care, and on other healthcare organizations’ services emerged from interviews, but the main findings of the study centered on intra-organizational context with potential to affect healthcare quality and actions for its improvement, including material resources, work organization design, teamwork, coordination and communication, professional responsibility vis-à-vis the patient, and leadership. A major issue was the divergence of physicians' and nurses' perspectives on care quality, which in turn negatively affected their capacity to work together.

**Conclusion:**

Overall, the findings suggest that care on the maternity hospitals was fragmented and lacked continuity, putting at risk the quality. Redesigning work organization, promoting conditions for multi-professional teamwork, better communication and coordination, improving more systemic accountability/lines of authority, and investing in team members’ technical competence, and fitness of organizational structures and processes are all imbricated actions that may contribute to obstetric care quality improvement.

**Electronic supplementary material:**

The online version of this article (10.1186/s12884-018-2088-3) contains supplementary material, which is available to authorized users.

## Background

Maternal morbidity and mortality are still serious public health concerns in Brazil. Millennium Development Goal (MDG) 5, which set a target of reducing maternal mortality by 75% between 1990 and 2015 to a maximum of 35 maternal deaths per 100,000 live births, was not achieved [1]. At the end of this period, maternal mortality in Brazil was 62/100,000 live births, 5–15 times higher than rates in high-income countries [2, 3].

Access to quality obstetric care is one essential pillar of efforts to achieve MDG 5 [4]. In Brazil, obstetric care is predominantly hospital-based and led by physicians [5], with high rates of medical intervention. The country has one of the highest cesarean rates in the world; of all births 52% occur by cesarean section, this rate being 43 and 88% in the public and private sectors, respectively [6, 7]. Despite ongoing efforts driven by the Brazilian Ministry of Health to lower rates of cesarean section and other obstetric interventions and to increase use of evidence-based practices for vaginal delivery in low risk pregnant women [8, 9], obstetric care quality problems and sub-optimal outcomes persist.

Much remains to be done to improve the quality and safety of obstetric care in Brazilian public hospitals. Efforts at multiple levels of the health system will be necessary [10], and one component must be quality improvement interventions at the ‘sharp end’ which aim to improve specific services. Successfully implementing such interventions remains formidably challenging, however.

Context is often a critical factor in the success – or failure – of improvement efforts to achieve desired gains in quality and safety [11–13]. Context affects determination of the appropriate priorities and targets for improvement interventions, while also furnishing barriers and facilitators of the implementation process. Optimizing local improvement efforts is thus likely to be supported by locally driven research that can inform the development and adaptation of appropriate evidence-based interventions and context-sensitive implementation processes [10]. The study reported here was carried out with this aim: embedded in a larger, multi-site project oriented towards developing and implementing an obstetric care quality improvement intervention in Brazilian maternity hospitals, this study aimed to identify contextual elements that would potentially affect the initiative.

Context includes both intra-organizational and extra-organizational features [11, 14]. Extra-organizational context can include, for example, wider health system policies or structures as well as fiscal conditions that impinge on organizations’ resources and infrastructure. Within organizations, adequate facilities and resources, as well as sufficient, skilled human resources are essential to high quality maternity/obstetric care [10]. Existing research also emphasizes that adequate resources are necessary but not sufficient: the complexity of services such as maternity care demands mechanisms to support coordination and mobilization of many inter-dependent resources and processes, as well as effective interpersonal communication and teamwork. Contexts characterized by a lack of ‘organizational fitness’ (weak operational systems) and/or poor inter-professional working relations pose significant challenges for achieving high quality care [15, 16]. Both the technical and the cultural aspects of organizational contexts matter [17].

Healthcare workers are a valuable source of intelligence about the challenges and opportunities for improvement, and the contextual features that well-designed interventions must take into account [12, 18]. This study used qualitative interviews with diverse healthcare professionals in three maternity hospitals to understand the challenges to delivering safe, high quality obstetric care, with a view to informing contextually-sensitive improvement interventions.

## Methods

The qualitative study was conducted in three public maternity hospitals of a large Brazilian city. The three units were nominated by the city’s Health Department to take part in the obstetric care quality improvement initiative, based on annual volume of births and buy-in from high-level managers. These units are among the five highest volume maternity units in the city – about 5000–6000 births annually each. They provide antenatal care to high-risk pregnant women, and the deliveries may be assisted by doctors as well as by obstetric nurses, depending on the level of risk involved. All have neonatal intensive care units; one has an adult intensive care unit available for mothers. Their cesarean rates are about 30–35%, lower than both the national mean and the Brazilian public sector mean.

Empirical data were obtained through individual interviews with healthcare professionals, conducted from July 28 to October 14, 2015 over the course of 12 site visits. Consolidated criteria for reporting qualitative research (COREQ) guidelines were followed (Additional file 1).  Thirty interviews were planned, 10 in each maternity hospital, based on sample specificity, study scope and existence of established theory [19]. Interviews were conducted by public health researchers – MCP (PhD), SMLL (MD, DSc) and LGCR (MD, DSc) – with additional qualitative research skills training and support provided by ELA (PhD). Having obtained informed consent from participants, interviews were conducted in private space in the hospitals. Using a semi-structured interview guide, questions sought to explore teamwork, coordination and communication, and leadership, being open to capture other contextual elements that could emerge (Additional file 2).

In each maternity unit, participants were purposively sampled to incorporate diverse perspectives: frontline staff vs ‘blunt end’ staff; seniority and responsibility (e.g. team leaders and nursing coordinators), specialism and/or discipline (e.g. physicians and nurses of different specialties such as obstetricians, pediatricians, anesthesiologists, obstetric nurses, generalist nurses), work patterns (e.g. staff who worked weekend as well as various daily shifts). We also sought to include participants who had at least 6 months experience of working within the maternity units. Following discussions with senior leadership from each hospital to establish interest and willingness to participate in the study and subsequent intervention, the research team work with local collaborators to facilitate data collection. Potential interviewees were invited face-to-face, after mediation of these collaborators, with researchers stressing that participation was voluntary and ensuring no information was shared with supervisors/local collaborators.

Interviews were audio-recorded and transcribed. Data were analyzed to identify features with potential to affect healthcare quality and improvement following the constant comparative method [20], supported by NVivo®11 software. Transcripts were recursively read and coded by MCP and SMLL. Following initial open coding, codes were organized into thematic categories through an iterative process of comparison across interviews and sites, informed by the conceptual framework described above. Cases of disagreement were discussed and resolved by consensus.

## Results

Twenty-seven interviews were carried out, ranging between 23 and 117 min, with a mean duration of 53 min. They involved 5 physicians and 3 nurses in formal leadership positions, 7 obstetricians, 2 anesthesiologists, 3 neonatologists, 5 obstetric nurses, and 2 general nurses.

Extra-organizational context concerning the dependence of the maternity hospitals on primary care units, responsible for antenatal care, and on other healthcare organizations’ services emerged from interviews, which are briefly presented. The main findings of this study centered on intra-organizational context with potential to affect healthcare quality and actions for its improvement.

### Extra-organizational context

Participants felt that some of the challenges experienced within maternity units stemmed from failures in other related services. Antenatal care was frequently criticized and evaluated as a determinant of poor outcomes observed among women and babies. Relating to antenatal care’s challenges the staff have highlighted women without antenatal care or who were poorly informed about, for example, their risk conditions, antenatal care performed by nurses and doctors without specialization in obstetrics, inadequate risk classification, and failure of primary care workers to comply with the care protocols.

Lack of coordination and formal communication with other healthcare organizations on whose services maternity units relied was also highlighted. For example, interviewees underlined concerns with prompt acquisition of blood and the transfer of women to an intensive care unit. The absence of a blood bank was especially felt in one of the maternity hospitals with more difficult access routes from alternative blood suppliers.

### Intra-organizational context

#### Material resources

At the time of the study, according to participants, the maternity hospitals had good structural conditions, with sufficiency and desirable diversity of professionals, and largely adequate physical installations, equipment, and diagnosis and treatment services. In some cases, participants indicated shortage of nursing staff or physicians, but it did not appear as a critical problem in fact. However, reflecting the challenges created by dependence on other organizations to provide certain services, staff perceived absence of those services within their own units (e.g. blood banks) as a deficit in the unit’s structure. For example, staff in the two hospitals that lacked a maternal intensive care unit argued that this undermined their ability to provide quality care given the patient profile, which included socially and economically vulnerable population and high-risk case mix.

Staff also reported problems of organizational fitness – that is, operational systems and processes that did not function effectively and efficiently Operating rooms, were not always replenished and ready for immediate use as would be expected in an emergency hospital.

*… We have had trouble with requesting blood for years… because people do not do it properly… “Where’s the blood? They said they could not send it because the hematocrit is missing … again? Who asked for this blood?” Then, you are at a loss as to why three people made the request…* [Doctor 05]

*So … I come in to perform a cesarean section … I open the drawer to get the material and it’s not there… it is inadmissible for a surgical center like this not to have a stock replenishment.* [Doctor 05]

#### Work organization design

Schedules and division of work across teams and over time contributed to a sense that care on maternity wards was fragmented and lacked continuity. Staff reported that it created an impression of being a different hospital every day among professionals (and even patients). Staff felt that this lack of continuity and consistency undermined quality of care, in addition to the risks inherent in hand-offs between shifts. The shift pattern was felt to reduce individual worker’s sense of accountability for patients, while the lack of alignment between nurse and physician schedules was perceived to limit cohesion – setting the professions apart.

*It does not exist, they are worlds apart, because, in fact, as nursing does not accompany the (medical) team due to the work schedule; Tuesday’s (medical) team, the nursing team works on Tuesday this week and works on Thursday next week…* [Doctor 12]

Participants also perceived difficulties in interactions between internal services, even when these services had clear roles defined, affecting timely responses to demands and/or resulting into overlap and wasting of resource and time. For example, it was registered, at least in one of the maternity hospitals, that there was no protocol for demanding the urine 24-h urine protein test, making timely measurement impossible during the night.

#### Teamwork

In addition to the constraints on multi-professional teamwork imposed by fragmented work schedules, teamwork difficulties were also associated with competing logics of care across the disciplines. Some professionals referred to the multidisciplinary group of professionals involved in patients’ care as a ‘team’. However, it was still common for nurses to consider that their team consists only of nursing professionals (higher and technical level), and for doctors to refer to their team as including only their medical peers – anesthesiologists, obstetricians and pediatricians.

*I can speak for my team… Our relationship with pediatrics, obstetrics and anesthesia is wonderful, it is a very integrated and united team …* [Doctor 09]

*I believe that the strong point within the hospital is the obstetric nursing team itself… the whole group is … engaged, participatory.* [Nurse 03]

Physicians and nurses highlighted the different logics espoused in medical and nursing education, which in turn shaped their performance and lead them to carry divergent childbirth care models. Medical training was perceived as essentially interventionist, with physicians tending to conduct labor and childbirth, and possessing the knowledge and skills for decision-making on diagnostic and therapeutic procedures in the occurrence of any complications. On the other hand, the training of nurses was pointed out as being more focused on the use of techniques to support women during normal and low-risk labor and delivery, which, from their perspective, was an exclusive process of women which should be conducted by women.

*As doctors, we are interventionists; we accelerate delivery… For example, this baby I delivered now, a 4-kilogram delivery, 3,995kg, I put forceps and I shortened that expulsive period… To others, for this line of … of humanized care people, I was interventionist, I did not think twice…* [Doctor 01]

*Obstetric nurses do not like intervention. Therefore, if they are following a delivery, this delivery will be without serum and analgesia; this patient will be walking, or sitting on a ball, horse or chair.* [Doctor 03]

The coexistence of these divergent perspectives often generated conflicts, and negatively affected the capacity of physicians and nurses work together and recognize themselves as being on the same team. This ‘cultural’ division was reinforced by the existence of two distinct chains of command, whereby nurses reported to a nursing coordinator and doctors reported to a physician coordinator. The articulation between these coordinators is essential to the establishment of effective teamwork, and when it is poor, all work and outcomes are compromised. Although staff recognized that timely and effective intervention required teamwork, the competing logics and fragmented lines of authority and accountability were felt to limit the integration of the knowledge and skills each offered.

In addition to relational issues, participants also raised concerns about technical qualification of nursing professionals and physicians, undermining respect and confidence.

*However, we are facing another issue, namely, training, bad and poor training. Now, are we preparing this professional properly to help reduce these indicators?* [Nurse 08]

*For example: there was a patient here this year … this employee is no longer working with us… but, a teenager came in with a 15/10 pulse, so. On admission, we need to pay close attention to this. The patient had a seizure on admission, thus, maximum alert on, and instead of treating eclampsia, which was obvious, he asked for an opinion from neurosurgery. This officer is no longer with us due to this technical difficulty, because, for me, that was basic stuff.* [Doctor 04]

Physicians believed that nurses delayed requesting physician intervention/support, either because they were not adequately trained to identify the risk or changes in the risk level, or because they wanted to show they could handle situations autonomously. For their part, nurses complained about doctors’ delayed responses when they were called.

*Thus, you have to be around … The nurse will not know when to call you. She will usually call you after the damage is done…* [Doctor 01]

*No! Today is not my turn. “X” is supposed to be on-duty on the ward now. “Go and call him.” – Well, if he is there, regardless of whether he is on duty or not, if you are calling, he has to go… Regardless of whether or not he is in the sector, if you ask … However, doctors do not always have such good will, so to speak.* [Nurse 01]

*What happens here with the normal birth? The nurses self-challenge and end up delivering a birth that is not theirs, while at the same time the doctor say the following words when they are called in. –“What? You don’t know how to perform a delivery?” Actually, she knows how to do it, but childbirth complications and unusual situations are part of medical training.* [Doctor 12]

Physicians’ statements also underline the aspect of legal liability for adverse events, a warning that the obstetric nurse can perform normal birth at regular risk, this does not exempt the physician from monitoring the development of labor and delivery itself.

*… I say to my colleagues that the responsibility inside is ours (medical) even when we have nursing around, because I sign the death certificate, not them… Any incident with any complications is my responsibility, so I refuse… I must oversee…* [Doctor 14]

#### Coordination and communication

In the interviews, the lack of written and oral communication among professionals of a team and among different teams stood out. Our findings also suggest poor communication between professionals and patients. Participants perceived communication as poorly structured, imprecise, and sometimes incomplete. Medical records and partographs charting patients’ progress, and common area white boards summarizing patient records were frequently not used, or only used by some team members but not others.

*If I say that all the shifts complete partographs as they should, I would be lying to you.* [Doctor 02]

For a professional to have a picture of all the various services rendered to a patient, he/she would have to resort to several records, which could not be obtained from a single source, even with a single electronic medical record system. This shortfall in written records was aggravated by lack of precision and timeliness that characterized oral communication between professionals, between teams, and between professionals and patients.

*… I always consult the electronic medical record … but I always check whether the person wrote it with a pen… what nursing wrote … I look at everything …* [Doctor 14]

Participants underlined that the relay of information about patients at handoffs most of the time occurred totally in parallel between nursing teams and between medical teams, compromising the possibility of a broader and multidisciplinary view of the cases to be followed up in the new shift. In addition, they also pointed out the lack of multi-professional rounds, as an initiative able to propitiate a more comprehensive view of patients’ cases. Some existing discussion meetings tended to reproduce the separation of professional categories.

*Doctors exchange shifts between them … and nursing, namely, the day shift. I arrive and she is waiting for me and tells me what is happening…* [Nurse 05]

*Thus, it lacks a “round” of professionals to discuss the patient’s case. I go in as a nurse; someone else goes in as a doctor, an obstetric nurse. I think this is missing, this discussion about the patient’s case! [Nurse 02].*

Moreover, interviewees associated poorly structured communication and slow responses with discontinuity of care, potentially worsening the patient’s clinical condition, and indicated problems of communication and coordination between maternity unit personnel and other support services, such as clinical analysis laboratories, pharmacy and so forth.

*There is a patient on the X floor with hypertension … The floor nurse calls the on-duty team on the Y floor … the person who answers the phone is usually a technician or a nurse … If the doctor is not there for a while … Folks, tell the on-duty person that we have a patient with hypertension here, all right? The information gets lost, and, sometimes, that one hour made the difference, because you only find out that the patient was having peaks three hours later, right?* [Doctor 04]

*Our priority is the blood typing of the mother and the baby, VDRL and HIV. The patient must undergo an HIV test before breastfeeding: do you think they have this priority? No! I have to call the lab five hundred times to find out whether the patient is HIV-negative so that she can breastfeed.* [Doctor 03]

#### Professional responsibility Vis-à-Vis the patient

Problems underlined above that undermined teamwork and created antagonism between professionals were also appeared to risk shrinking individual professionals’ sense of personal responsibility, promoting instead a tendency toward non-cooperation and shifting of responsibilities onto other professionals. Norms and standards on maternity wards are sometimes used as an argument – “this is not my responsibility” – so that no one were responsible for solving problems in effective patient care.

*For example, the ultrasound doctor arrived, she has to check on the patients on the floor; we have scheduled visits and those of the emergency room… One says: “I have no obligation to take the patient” and the other one replies: “I have to call in the stretcher bearer”, “but the stretcher guy went to the blood bank…” “That’s not my job, that's the guy’s job!” Well, there’s only one guy out there… [LAUGHING]* [Doctor 04]

#### Leadership

Participants of this study expressed their expectations about attributes of leaders. They have expected that leaders would be able to manage conflicts and to facilitate team-building and harmonization. It was also expected that leaders would have technical competence, listening skills, command ability for assignment of roles among team members, as well as capacity to implement actions to ensure coordination and integration of care. Thus leadership, whether they like it or not, seems works as an example to be followed, thereby, their behavior, attitudes and daily presence had an impact on the behavior of the teams and each professional.

*… so that person must have a profile of mediator, of someone able to listen, and after having heard, provides a response on what he/she has heard, go there at the other end, which was the object of the complaint and check what happened, listens to that party as well and, after hearing both parties, enters into an understanding and take it to those two conflicting parties, right? I believe that the leader must have this profile, he/she shouldn’t be that explosive person… he/she has to be a quiet person with the ability to listen, someone who has the ability to talk to the other person in a non-aggressive way and knows how to coordinate things and assign tasks.* [Doctor 14]

Interviewees demonstrated the recognition of existing leaderships on the maternity wards, either at the general management level or at shifts, and have stated that they help to improve the patient records, team performance, and in articulating internal and external actions for adequate care provision. However, they also identified the existence of formally appointed leaders who were not prepared to take this role effectively, as well as others who were very centralizing and ultimately made teams dependent. Participants recognized the difficulty of identifying professionals with all the desirable qualities and who are willing to accept additional leadership responsibilities. It is worth commenting that the interviewees also pointed out the existence of professionals who had a leading role, either by characteristics of proactivity or by recognition by colleagues about their knowledge and professional competence. In fact, there was a paradoxical perception on leadership acting. Without an actual position in the organizational decision-making, the role of these informal leaders was sometimes singled out as positive, but by others as negative for coming up against the formal leader.

Briefly, the whole set of elements presented points out the need of leaders, at the organizational level, but, especially, at the healthcare microsystems level in the teams, able to tackle conflicts, to coordinate care, and to face other challenging problems in the maternity hospitals.

## Discussion

In general terms, the challenges to delivering safe, high quality obstetric care identified in the maternity hospitals involved in this study, correspond, at the organizational level, to those often indicated in the literature [21, 22]: issues concerning work organization design, bad communication and coordination, teamwork and leadership. Material and human resources were not indicated as a major problem at the time of the interviews, emerging from them, on the other hand, problems concerning the low level of responsibility for the patient assumed by frontline professionals in their fragmented view of patients and care processes.

In more specific terms, however, different points may be raised under the labels referred. Teamwork, for example, seemed affected by the work organization design, including features such as on-call schedules, and shift pattern, as well as by professionals’ training and divergent perspectives on how to care. A major issue was the apparently still in-process assimilation of a relatively new model of obstetric care in which obstetric nurses came to assume the conduction of low risk childbirth care, a role before attributed to obstetric physicians. Power struggle, some degree of distrust and little mutual respect finished undermining what could otherwise be a desirable form of complementary work. Two models seemed to coexist – one of the doctors and another of the nurses. These professionals did not seem to have been able to establish a single integrated model of childbirth care, in which their roles were clearly defined and complementary.

The capacity to coordinate work in the maternity hospitals was also latent. In the intra and extra organizational context, it was expressed in the disarticulation between internal services, intra- and inter-teams and in the relationships of the maternity units with the healthcare network. Despite the efforts that have been undertaken to facilitate a better articulation between the primary care units responsible for providing antenatal care and the maternity hospitals, it was evident, in the voices of participants of this study, that there were problems in the services’ network that affected the work in the maternity hospitals.

While results of this study indicated the use of more formal work coordination devices, such as care protocols and team meetings (rounds) or less formal mechanisms, there still seems to be an imbalance or even insufficient use, depending, among other things, on shifts. Perhaps one of the most valued mechanisms is the organization of rounds among team members, including doctors and nurses. On the one hand, they help build new knowledge and skills and, on the other, provide an effective opportunity to exchange information and strengthen face-to-face communication [23]. They could also contribute to the generation and accumulation of trust among team members, a central condition to teamwork development and valorization of mutual respect.

Collective team discussions could help better understand responsibilities and adjust expectations among members, who need to develop the capacity to deal with situations/problems for which they do not have ready-made answers, a priori, and to exercise the ability to open one’s own mind to new solutions [23]. Perhaps the precise role demarcation is difficult and, therefore, requires each professional to be more tolerant in addressing ambiguous situations, without losing sight of preparedness, quality and safety of the care provided to patients. In these cases, the patient-centered care process must be the reason of the professionals on duty, making teamwork prevail.

Brazilian efforts to incorporate nurses who specialize in the labor and delivery assistance of low-risk pregnant women have been inspired by the successful experience of midwives in the United Kingdom [24–26]. However, the Brazilian context is completely different from that of the UK, so there are still many challenges to be faced, such as professional classes’ interests, training deficiencies, and other relevant barriers. This new implementation context may impose adaptations, and its analysis is important to clarify relevant elements or difficulties that, if not carefully considered, can compromise the expected outcomes [27].

A powerful initiative to address the problems listed are continuing education strategies – specific technical training, use of protocols, effective communication, teamwork – such as simulations or other types of in-service training [23]. Specialized training enables the development of specific knowledge and skills, but also hinders integration of the various contributions in the delivery of care. So that means it encourages the perception that the quality of care is much more dependent on knowledge and individual work than on the coordination of own work with that of other professionals [28]. Such training, in turn, leads to different perceptions about the same problem and ends up compromising communication among professionals [23].

Professional training shortfalls were highlighted, and it is important to consider the need for greater interaction with the Universities, and training and regulatory organizations of the mid-level and higher-level professionals involved. Technical competence is strongly valued in building trusting relationships for teamwork [29]. At the same time, issues such as teamwork, coordination of services and communication are still insufficiently incorporated in the curricula [21–23].

It is also fundamental to identify, qualify and empower leaders to perform organizational adjustments, whether in a more operational scope or in the behavioral realm, with a view to offering safe, timely, quality obstetric care centered on women and their babies [23].

Finally, a major challenge is a cultural shift towards introjection of the patient-centered care, defined as “the provision of care in a respectful way, responding to the needs, preferences and values of the person under care, with the assurance that such values guide all clinical decisions” [30]. The fragmented care processes and accountability restricted to specific processes (“to each his own”) compromised all quality of care dimensions. Patient centrality can be a catalyst for the continuity of care and interprofessional collaboration, also pointed out with central elements in the provision of obstetric care [31].

We did not interview nurse technicians, despite the recognition of their relevant role in the obstetric care settings in the study. They could add to the findings other elements concerning work dynamics and hierarchical relations in the maternity units. Moreover, this study was not intended to provide generalizable findings. In spite of that, it certainly ratifies issues that still mark the supply of obstetric care in different contexts around the world [28, 31–33]. As such, while inclusion of further sites may have identified additional contextual influences, the dynamics and challenges identified in the hospitals in this study may be judged transferable and resonant in similar settings [34]. Dealing with the observed elements is crucial for any interventions aimed at improving obstetric care.

## Conclusions

Overall, the drafted picture provided a sense that care on the maternity hospitals studied was fragmented and lacked continuity, putting at risk the quality. Although specific intra-organizational issues were detached, they are, in fact, deeply imbricated with each other. Teamwork, and individual and collective responsibility for patient care, for example, are themselves undermined by work organization design, lack of coordination, and other elements. Therefore, redesigning work organization, promoting conditions for multi-professional teamwork, better communication and coordination, improving more systemic accountability/lines of authority, and investing in team members’ technical competence, and fitness of organizational structures and processes are all imbricated actions that may contribute to obstetric care quality improvement.

Additionally, the findings expose tensions that still mark the incorporation of the model of childbirth care conducted by obstetric nurses in maternity hospitals in Brazil, which coexists with the more traditional and interventionist model conducted by obstetric physicians with low integration, lacking the desirable complementarity. The new model has been an effort to revert the high rates of unnecessary obstetric interventions related to the traditional model and was inspired in the successful UK obstetric care model. However, the adaptation of successful interventions to other contexts imposes its own challenges given contextual differences.

## Additional files

Additional file 1: COREQ checklist. The file contains documentation on compliance of the study/manuscript with COREQ guidelines. (DOCX 22 kb)

Additional file 2: Interview guide. The file contains the interview guide employed in the study. (DOC 424 kb)

## Abbreviation

MDG: Millennium Development Goal
