# Supplementary material for: Challenges to the improvement of obstetric care in maternity hospitals of a large Brazilian city: an exploratory qualitative approach on contextual issues
Source: BMC Pregnancy Childbirth. 2018 Nov 26;18:459. doi: 10.1186/s12884-018-2088-3 (PMC6258487; doi:10.1186/s12884-018-2088-3)
Supplement: Supplementary file 1 — COREQ checklist. The file contains documentation on compliance of the study/manuscript with COREQ guidelines. (DOCX 22 kb) [file 12884_2018_2088_MOESM1_ESM.docx]

[View Large](https://academic.oup.com/view-large/%5bXSLTSectionID%5d)

**Table 1**

Consolidated criteria for reporting qualitative studies (COREQ): 32-item checklist

| **No** | **Item** | **Guide questions/description** |
| --- | --- | --- |
| **Domain 1: Research team and reflexivity** |  |  |
| Personal Characteristics |  |  |
| 1. | Interviewer/facilitator | Which author/s conducted the interview or focus group?  **Methods, paragraph 2** |
| 2. | Credentials | What were the researcher's credentials? *E.g. PhD, MD*  **Methods, paragraph 2** |
| 3. | Occupation | What was their occupation at the time of the study?  **Methods, paragraph 2** |
| 4. | Gender | Was the researcher male or female?  **Indicated by authors’ names; no basis to consider this a crucial identity dimension in the interpersonal relationship given the professional context and subject of interviews** |
| 5. | Experience and training | What experience or training did the researcher have?  **Methods, paragraph 2** |
| Relationship with participants |  |  |
| 6. | Relationship established | Was a relationship established prior to study commencement?  **Methods, paragraph 3** |
| 7. | Participant knowledge of the interviewer | What did the participants know about the researcher? e*.g. personal goals, reasons for doing the research*  **Additional_file_2 – Interview Guide, paragraph 1** |
| 8. | Interviewer characteristics | What characteristics were reported about the interviewer/facilitator? e.g. *Bias, assumptions, reasons and interests in the research topic*  **Role and qualifications of interviewers reported in methods section, paragraph 2.**  **Motivating interest in research topic reported in background section, paragraph 6 and methods section para 1.** |
| **Domain 2: study design** |  |  |
| Theoretical framework |  |  |
| 9. | Methodological orientation and Theory | What methodological orientation was stated to underpin the study? *e.g. grounded theory, discourse analysis, ethnography, phenomenology, content analysis*  **Conceptualization of context (as relevant to quality improvement) is described in background section, paragraphs 5-6.**  **Methodological orientation (Charmaz’s constant comparative method) described in methods section, paragraph 4.** |
| Participant selection |  |  |
| 10. | Sampling | How were participants selected? *e.g. purposive, convenience, consecutive, snowball*  **Methods, paragraph 3** |
| 11. | Method of approach | How were participants approached? e*.g. face-to-face, telephone, mail, email*  **Methods, paragraph 3** |
| 12. | Sample size | How many participants were in the study?  **Results, paragraph 1** |
| 13. | Non-participation | How many people refused to participate or dropped out? Reasons?  **No one actively declined, but the Director of Nursing of one of the maternity units was not interviewed. We tried scheduling the interview with her sometimes through our local collaborators, and we could perceive some difficulties. At some point she was fired.**  **Additionally, we did not interview nurse technicians, an important staff category in the maternity units – discussion section, paragraph 11.** |
| Setting |  |  |
| 14. | Setting of data collection | Where was the data collected? e*.g. home, clinic, workplace*  **Methods, paragraph 2** |
| 15. | Presence of non-participants | Was anyone else present besides the participants and researchers?  **Methods, paragraph 2** |
| 16. | Description of sample | What are the important characteristics of the sample? *e.g. demographic data, date*  **Sample characteristics pertinent to this study are site and professional role: results section, paragraph 1.**  **Data collection period: methods section, paragraph 2.** |
| Data collection |  |  |
| 17. | Interview guide | Were questions, prompts, guides provided by the authors? Was it pilot tested?  **Additional_file_2 – Interview Guide** |
| 18. | Repeat interviews | Were repeat interviews carried out? If yes, how many?  **N/A** |
| 19. | Audio/visual recording | Did the research use audio or visual recording to collect the data?  **Methods, paragraph 4** |
| 20. | Field notes | Were field notes made during and/or after the interview or focus group?  **Field notes were not made. Interviews were audio recorded.** |
| 21. | Duration | What was the duration of the interviews or focus group?  **Results, paragraph 1** |
| 22. | Data saturation | Was data saturation discussed?  **No. Malterud et al.’s (2015) concept of information power informs sample size. Methods, paragraph 2** |
| 23. | Transcripts returned | Were transcripts returned to participants for comment and/or correction?  **No.** |
| **Domain 3: analysis and findings**z |  |  |
| Data analysis |  |  |
| 24. | Number of data coders | How many data coders coded the data?  **Methods, paragraph 4** |
| 25. | Description of the coding tree | Did authors provide a description of the coding tree?  **No, but process of analysis is explicitly described. In line with constant comparative method, the codes evolved over iterative passes through the data. The structure of the results section describes the resulting themes, using illustrative quotes, reflecting the ‘coding tree’.** |
| 26. | Derivation of themes | Were themes identified in advance or derived from the data?  **Methods, paragraph 4** |
| 27. | Software | What software, if applicable, was used to manage the data?  **Methods, paragraph 4** |
| 28. | Participant checking | Did participants provide feedback on the findings?  **Findings were presented in the maternity units. In general, there was broad agreement and enthusiasm to use the findings to inform intervention.** |
| Reporting |  |  |
| 29. | Quotations presented | Were participant quotations presented to illustrate the themes / findings? Was each quotation identified? e*.g. participant number*  **Yes.** **Throughout results section.** |
| 30. | Data and findings consistent | Was there consistency between the data presented and the findings?  **In our view, yes. Throughout results section.** |
| 31. | Clarity of major themes | Were major themes clearly presented in the findings?  **Yes, as reflected in subheadings throughout results section.** |
| 32. | Clarity of minor themes | Is there a description of diverse cases or discussion of minor themes?  **Diversity and variation in views or salience of themes across participants or site is reported where relevant throughout results section, (eg. teamwork, paragraph 1-2)** |
