# Supplementary material for: Challenges to the improvement of obstetric care in maternity hospitals of a large Brazilian city: an exploratory qualitative approach on contextual issues
Source: BMC Pregnancy Childbirth. 2018 Nov 26;18:459. doi: 10.1186/s12884-018-2088-3 (PMC6258487; doi:10.1186/s12884-018-2088-3)
Supplement: Supplementary file 2 — Interview guide. The file contains the interview guide employed in the study. (DOC 424 kb) [file 12884_2018_2088_MOESM2_ESM.doc]

**Development and evaluation of a multifaceted quality improvement intervention for obstetric care in maternities in Rio de Janeiro**

***Frontline professionals’ Interview Guide***

**Introduction of research by the interviewer to the interviewee**

[The interviewer must provide an overview of the research-intervention to the interviewee. She/he must also indicate the interest of the interview in capturing his/her impressions about the maternity context with regard to initiatives/concerns and environment to healthcare quality improvement and patient safety, to work environment, to relationship with patients and their families, to healthcare provided and to how emergency situations are dealt by the healthcare professionals. Before the beginning of the interview, the Consent Form must be signed in two copies, one for the interviewee and the other to the research principal investigator].

**Questions and some supportive appointments**

1. **To begin, could you start by telling me about your role in this maternity, what kind of contract you have and how long you’ve worked here?**

**- do you work anywhere else?**

1. **Thinking about quality, safe care for patients, what would you say are the strengths of this maternity?**
2. **And what are your main challenges? What problems do you face trying to provide safe, quality care?**
3. **Frequently, medical records are criticised because of their incompleteness and inadequacy for the coordination and continuity of patients’ care. Do you identify any difficulty in this sense in this maternity?**
4. **Would you say that communication among the members of your team and between your team and others is adequate for care coordination?**
5. **How would you describe the working relationships between professionals here?**

**- amongst nurses?**

**- between doctors and nurses?**

**- would you say you all work as a team’?/ does it affect coordination of care?**

1. **Do you think that it is important the presence of leaders in the healthcare process. For you, who are these leaders and which role do they play or should they play?**

- **formal structures**
- **informal leadership**

1. **How would you describe this maternity’ patients? In which extent do you believe it is appropriate to consider their preferences and to involve them and their families in the care process?**
2. **Among the United Nations’ millennium goals, the maternal mortality was the only not reached by Brazil in 2015, where high rates are observed even in the reality of Rio de Janeiro City. On the other hand, there have been a hot debate about the excess of caesarean sections in the country. How do you see these questions, and, from your point of view, how should this maternity deal with them?**
3. **Have any improvement efforts been tried already? How well (or not) did they work?**
4. **If you had to propose healthcare quality improvement interventions in this maternity, what would you propose?**
5. **Which factors, in your opinion, may facilitate or make difficult the implementation of healthcare quality improvement interventions in this maternity?**
6. **If a friend or family member of yours was coming here to have their baby, would you have any concerns? What would you be most concerned about?**
